# Our City Noises

**Published:** 1906-10-06

**Authors:** 


### Our City Noises.

Modern civilisation is sometimes reproached with having made men more sensitive, and certainly this is not an age of hair-shirts. But if modern man is more susceptible to the influence of external sense-stimuli than his ancestors, it is very unfortunate that he should have to endure impressions, which for number, variety, and intensity could not, surely, be matched in any previous age. Our civilisation is, above all things, noisy. It is doubtful if the feelings of a hardy baron of the Middle Ages, condemned to wait half an hour in a large railway terminus, could receive adequate expression in any language of modern days. We venture to think that at the end of the time the unfortunate warrior's nerves would be in an unsettled condition. The unending clatter and rustle of a thousand feet, the recurrent ear-splitting shriek, and a multitude of other noises, combine to produce a deafening din, such as no medieval clamour could have equalled. Add to that the newsboy's obbligato, the metallic clatter of milk-cans thrown upon a platform by careless porters, might have suggested to Dante an eighth circle of the Inferno.

In our city streets the baron would fare no better. The incessant tramp, yells of newsvendors, rattle of buses, roar of motor-cars and trams, would distract him and pursue him to his home. No medieval castle, were the walls never so thick, could shut out the penetrating *jödel* of the milkman, the insistent fortissimo noise of street pianos, and the wonderful variety of street cries. From early morn, when a brass band *en route* for Hampstead or Epping, crashes in upon our slumbers, till dewy eve, when the accordion goes to its home and drunkards bawl in the streets, a noisy civilisation belabours our auditory nerve-centres as on an anvil. There is no escape. The wakeful dog punctuates the midnight hours by yelping at timed intervals corresponding to the moment when the wearied brain is on the point of falling off into nothingness and constitutes one of the most irritating experiences that civilisation can produce.

No wonder that in these days quiet has become synonymous with rest. Probably no element of modern life has such a wearing effect upon the nervous system as noise. The constant succession of auditory stimuli, violent and sustained, to which is often added the element of suddenness, which induces that expensive discharge of nerve-energy known as "starting," produces after a time in many individuals a more or less complete exhaustion of the nerve-centres. A familiar instance of this kind of exhaustion everyone has experienced in the tiredness which follows upon a long railway journey. The initial excitement and worry of looking after luggage and baggage no doubt play their part in the production of the subsequent loss of energy, but probably the prime agent in the tiring process is the incessant roar of a train in rapid motion.

There is, of course, much variation in the manner in which different people react to such stimuli. Some favoured individuals seem absolutely immune from the evil effects of exposure to loud noise, just as some people are happily ignorant of sea-sickness. In others the weariness amounts to a degree of exhaustion, which, if frequently repeated, may lead to serious illness. It would not be overstating the case to ascribe much of the modern increase of insanity to our noisy environment. Consequently, for the sake of the mental and bodily health of the more delicate of our citizens, as well as for our own comfort, we should endeavour to moderate the quality of the noises modern flesh is heir to.

Some efforts are now being made to combat the evil. Legislation has put within the reach of us all power to interdict the continuance of objectionable noises, albeit only after a tedious process at law. One of the developments of the motor-car, in obedience to the new-born desire for quietness, is in the direction of silencing the engine, and the success obtained is interesting, as showing what can be done to quieten noisy machinery, when a serious attempt is made to do so. A Northern railway company has, if we mistake not, in a moment of public-spiritedness, substituted the low and not unmusical steamship horn for the piercing locomotive whistle. But much remains to be done before comfort or even safety is reached. The police should be able to suppress summarily street cries of all kinds, barking dogs, pealing church-bells, detonating motor-cycles, singers, itinerant musicians, and the whole legion of this Inferno of noises. In a city there always must be a large and varied assortment of more or less necessary noise, but were all unnecessary sounds stilled the lot of the city dweller would be infinitely more comfortable and healthy than at the present day, when, only between midnight and four in the morning does "silence, like a poultice, come, to heal the blows of sound."

